# Prolonged life-threatening hypoglycaemia following dose escalation of octreotide LAR in a patient with malignant polysecreting pancreatic neuroendocrine tumour

**DOI:** 10.1530/EDM-14-0097

**Published:** 2015-01-01

**Authors:** Sally K Abell, Jessie Teng, Anthony Dowling, Michael S Hofman, Richard J MacIsaac, Nirupa Sachithanandan

**Affiliations:** 1 Department of Endocrinology and Diabetes, St Vincent's Hospital, PO Box 2900, Fitzroy, Melbourne, 3065 Victoria, Australia; 2 Department of Oncology, St Vincent's Hospital, PO Box 2900, Fitzroy, Melbourne, 3065 Victoria, Australia; 3 Department of Medicine, University of Melbourne, Parkville, Melbourne, Victoria, Australia; 4 Molecular Imaging, Centre for Cancer Imaging, Peter MacCallum Cancer Centre, East Melbourne, Melbourne, Victoria, Australia

## Abstract

This paper details the case of a 77-year-old male with refractory hypoglycaemia due to inoperable metastatic pancreatic neuroendocrine tumour (pNET) co-secreting insulin and gastrin. Multiple medical therapies were trialled with limited success, and we describe the complications experienced by our patient. Somatostatin analogues can ameliorate hypoglycaemia and may have tumour-stabilising effects; however, in our case resulted in paradoxical worsening of hypoglycaemia. This rendered our patient hospital dependent for glycaemic support including continuous dextrose infusion. Although this is a reported adverse effect with initiation of therapy, we describe successful initiation of short-acting octreotide as an inpatient followed by commencement of long-acting octreotide. Hypoglycaemic collapse occurred only after dose titration of long-acting octreotide. We outline the pitfalls of somatostatin analogue therapy and the mechanisms that may contribute to worsening hypoglycaemia. This rare side effect cannot be reliably predicted, necessitating close supervision and glucose monitoring during therapy. Our patient achieved disease stabilisation and gradual resolution of hypoglycaemia with peptide receptor radionuclide therapy (PRRT), an emerging therapeutic option for metastatic neuroendocrine tumours with high efficacy and low toxicity. We present a brief but comprehensive discussion of currently available and novel therapies for insulin secreting pNETs.

**Learning points:**

- Hypoglycaemia due to malignant insulin secreting pNET is frequently severe and may be life-threatening despite supportive therapies.
- Octreotide can ameliorate hypoglycaemia, and may have anti-proliferative and tumour-stabilising effects in malignant pNETs that are surgically unresectable.
- Paradoxical worsening of hypoglycaemia may occur with octreotide initiation and dose titration, necessitating close supervision and glucose monitoring.
- PRRT is emerging as a therapeutic option with high efficacy and low toxicity.

## Background

Well-differentiated pancreatic neuroendocrine tumours (pNETs) are heterogeneous tumours with variable behaviour and response to conventional therapies. They have an estimated incidence of ∼1/100 000 individuals, and insulinomas represent up to one-third of functioning tumours (1). Surgery is the only curative option for those with isolated primary lesions or limited metastatic disease, but can also be considered for debulking of symptomatic disease. However, up to 65% of pNETs (2) and 10–15% of insulinomas (1) may have widespread metastases at diagnosis.

Symptomatic hypoglycaemia may be very difficult to control in malignant insulinoma. We report a case of recurrent inoperable metastatic pNET co-secreting both insulin and gastrin, with resultant complications of hormonal secretory syndromes. Our discussion focuses on insulin hypersecretion and the occurrence of frequent hypoglycaemia refractory to medical therapies. Long-acting somatostatin analogue therapy resulted in initial improvement but paradoxical worsening of hypoglycaemia with dose titration. Although this has been reported with dose initiation, we are not aware of any previous association with dose titration. We describe the possible mechanisms of somatostatin analogue related hypoglycaemia, pitfalls of somatostatin analogue therapy and need for close supervision during therapy. We discuss currently available medical therapies including new agents, with the main aims being tumour stabilisation and symptom control, rather than the traditional oncologic goal of disease remission.

## Case presentation

A 77-year-old man was referred to the Endocrinology Department at our hospital with metastatic well-differentiated polysecreting pNET, secreting gastrin and insulin. He initially presented 8 years before with Zollinger–Ellison syndrome (gastrin 1820 pmol/l, normal 6–55 pmol/l). He had no symptoms of hypoglycaemia at that time. He underwent curative intent distal pancreatectomy, left hemi-hepatectomy, splenectomy and cholecystectomy. Histology revealed a 40 mm well-differentiated NET of the pancreas and a 170 mm solitary hepatic metastasis. All margins were clear. Hormonal staining was not performed on this specimen. The Ki-67 proliferative index was 2%, consistent with European Neuroendocrine Tumour Society (ENETS) Grade 1 tumour. His gastrin level normalised post-operatively. Serum chromogranin-A was not available and no other neuropeptides were measured.

## Investigation

Recurrent disease was detected 3 years later with a rise in serum gastrin to 2974 pmol/l, and symptomatic hyperinsulinaemic hypoglycaemia confirmed by 72-h fast. A 37×36 mm left para-aortic soft tissue mass was localised on computed tomography (CT) scan and ^111^indium-octreotide SPECT/CT scan. Repeat surgical resection achieved biochemical remission and complete symptom resolution. The tumour stained positively for gastrin, but not for insulin.

## Treatment

Regular biochemical surveillance revealed a mild increase in gastrin and chromogranin-A 3 years later. Imaging showed low volume metastatic disease (T11 transverse process, para-aortic nodes and hepatic metastases), but due to the indolent behaviour this was monitored without treatment. However, 5 years following the second resection, he represented with frequent episodes of symptomatic hypoglycaemia suspicious for recurrent hyperinsulinism. CT scan of the chest and abdomen showed extensive hepatic metastases, low volume osseous disease and peri-aortic and portal lymphadenopathy. Serum gastrin (2395 pmol/l) and chromogranin-A (660 U/l, normal <21.8 U/l) were elevated. A 72-h fast was terminated prematurely due to hyperinsulinaemic hypoglycaemia with insulin 58 mU/l (normal <10 mU/l) and plasma glucose 2.8 mmol/l (C-peptide 1.91 pmol/l, normal <0.07 pmol/l and pro-insulin 776.4 pmol/l, normal <13.3 pmol/l). As an inpatient, his lowest capillary glucose levels were ∼2.3 mmol/l. Diazoxide and dexamethasone were initiated, with a diet of frequent complex carbohydrate meals. Subcutaneous octreotide was commenced as an inpatient with good effect, and titrated to long-acting octreotide (LAR) 20 mg monthly. Following discharge, his hypoglycaemia was less severe; however, he continued to have frequent episodes and octreotide LAR was increased to 30 mg.

One week after dose escalation of octreotide LAR, he was readmitted following an unconscious hypoglycaemic collapse. He had intractable hypoglycaemia, frequently having a capillary glucose level of <1.5 mmol/l. Therapy was rapidly escalated to include high-dose diazoxide, dexamethasone, hydrochlorothiazide and glycosade powder between meals. Nasogastric feeds were poorly tolerated. Octreotide LAR was discontinued. He continued to have episodes of hypoglycaemia necessitating a continuous 20% dextrose infusion. Regular intramuscular glucagon only provided transient relief. Treatment was complicated by 30 kg weight gain, fluid overload, and symptomatic hyponatraemia (Na 114 mmol/l) leading to discontinuation of diazoxide and hydrochlorothiazide. The presence of pneumonia and line-associated sepsis precluded the use of chemotherapy or everolimus. Therefore, he managed with dexamethasone 4 mg daily, enteral feeding and continuous 50% dextrose infusions.

Given his persistent unremitting hypoglycaemia and reliance on continuous intravenous dextrose infusions, a trial of peptide receptor radionuclide therapy (PRRT) with ^177^lutetium DOTA-octreotate (LuTate) was administered in combination with radiosensitising 5-fluorouracil (5-FU).

## Outcome and follow-up

The patient underwent four cycles of induction PRRT. The second cycle was complicated by an upper gastrointestinal bleed in the setting of persistent hypergastrinaemia, despite medical therapy with proton pump inhibitors and histamine antagonists. This was successfully treated with urgent gastroscopy, intralesional adrenaline and clipping of a large duodenal ulcer. The cycles were otherwise well tolerated. Blood glucose levels stabilised following the second cycle of PRRT, facilitating discharge from hospital with dexamethasone, frequent carbohydrate meals and nocturnal 20% dextrose feeds. Glycaemic supports were ceased 3 months post cycle four of PRRT with ongoing biochemical response. He has no symptoms of hypoglycaemia and remains off glycaemic support at 24 months follow-up, with a 75% reduction in chromogranin-A level. Imaging shows marked radiologic and scintigraphic disease regression.

## Discussion

Hypoglycaemia can be life-threatening in inoperable malignant insulinomas and is difficult to treat (1). The antihypertensive agent diazoxide is commonly used as a first-line therapy for its ability to inhibit insulin release from β-cells, through effects on the ATP-sensitive potassium channels (1). However, tolerability can limit its use, as in our patient, with side effects such as oedema, rash, nausea, dizziness and anorexia (1). Glucocorticoids induce hyperglycaemia by inhibiting insulin release and increasing peripheral insulin resistance (1). However, their long-term use for glycaemic support should be balanced against potential adverse effects. In our patient high-dose dexamethasone therapy was associated with significant weight gain without demonstrable benefit for hypoglycaemia.

Conventional chemotherapy for well-differentiated pancreatic NETs consists of streptozocin (with diabetogenic effects) in combination with doxorubicin or 5-FU (1). Recently the combination of capecitabine (an oral prodrug of 5-FU) with temozolamide (3) and targeted therapies such as everolimus (4) and sunitinib (5) have demonstrated anti-tumour efficacy in pancreatic NETs. Everolimus may also be effective in refractory hypoglycaemia by interrupting the insulin-signalling cascade and inducing peripheral insulin resistance (6). However, there are no guidelines supporting first-line use of targeted therapies (1), and these options were not suitable for our patient due to his poor performance status.

Well-differentiated NETs usually retain high somatostatin receptor (SSTR) expression. Of the five subtypes of SSTRs (named SSTR1–5), SSTR2 and SSTR5 are most commonly expressed in insulinomas (∼70%) (7). These tumours can be detected by octreotide scintigraphy and treated with radiolabelled or cold somatostatin analogue therapy. Lanreotide and octreotide predominately bind with high affinity to SSTR2 and to a lesser extent to SSTR5, and in one series of SSTR2-positive benign insulinomas, octreotide was effective in reducing hypoglycaemia in 50% of patients (6). Pasireotide, a novel somatostatin analogue with high affinity for SSTR1–3 and SSTR5 may have a future role (1). In addition to inhibiting insulin release and elevating blood glucose levels, somatostatin analogues also have anti-proliferative and tumour-stabilising effects (8). They are generally well tolerated; however, their efficacy in restoring euglycaemia in malignant insulinoma has not been fully elucidated.

Somatostatin analogues also inhibit the counter-regulatory hormones glucagon and growth hormone, which antagonise the effects of insulin (9). Therefore, they can aggravate hypoglycaemia paradoxically, as in our case, which may account for the apparent lack of clinical benefit seen in some cases (9) (10). Loss of SSTR2 expression on insulinoma cells has also been described (9); however, we do not feel this is a plausible explanation in our patient due to confirmed high SSTR expression on SSTR imaging repeatedly on several occasions. Decreased hepatic glycogen stores and gluconeogenesis due to malignant infiltration can also aggravate hypoglycaemia, and may have been contributory in our patient (10). Predicting response to somatostatin analogues can be challenging and neither octreotide test doses nor Indium-111 scintigraphy may be helpful in some patients (7). Hence, blood glucose levels should always be monitored closely when initiating therapy with octreotide or following dose escalation in all patients with malignant insulinoma. In our patient, protracted and life-threatening hypoglycaemia occurred following dose escalation of octreotide LAR, despite intensely octreotide-avid disease on imaging and tolerating subcutaneous octreotide dose titration as an inpatient. Unfortunately, we did not have any concurrent functional imaging at this point to determine disease status. However, chromogranin-A levels following commencement of octreotide were lower than previously measured (160 U/l vs 660 U/l pre-therapy). The clinicians involved felt that the temporal relationship of worsening hypoglycaemia was related to the octreotide effect rather than sudden worsening of underlying disease or simply treatment failure. Following discontinuation of octreotide LAR he continued to have recalcitrant hypoglycaemia for 2 weeks, which may relate to a plateau effect of octreotide which can produce clinical effects for up to 4 weeks after discontinuation, and possibly diminished glycogen stores also. It was unclear whether discontinuation of octreotide LAR alone resulted in recovery, as other rescue therapies were administered simultaneously.

PRRT using radiolabelled somatostatin analogues has recently been shown to improve symptoms and quality of life in patients with inoperable functional pNETs (11). As most well-differentiated metastatic pNETs express SSTR2 receptors (12), they may be targeted not only with ‘cold’ somatostatin analogues but also with PRRT using radiolabelled octreotide such as LuTate (12) (13). Lutetium-177 is a β emitter with mean path length of 1 mm, enabling delivery of high doses of particulate emission to sites of tumour whilst sparing normal tissues (12). Studies have shown LuTate therapy to result in good tumour response rates in a high percentage of patients with metastatic NETs, with low toxicity (12) (13). LuTate was trialled in our patient to reduce hormonal overproduction and stabilise disease. It resulted in gradual restoration of euglycaemia, which has been maintained for 2 years following his last treatment dose.

## Conclusion

We present a case of inoperable polysecreting pNET with metastases to liver, lymph nodes and bone, with severe symptomatic hypoglycaemia despite multiple therapies.

Octreotide is commonly used as a first-line therapy to ameliorate hypoglycaemia and control tumour growth in patients with malignant insulinomas that are surgically unresectable. However, paradoxical worsening of hypoglycaemia can occur during initiation or after dose escalation, mandating close blood glucose monitoring. In our patient, the hypoglycaemic response proved difficult to manage due to its refractory nature and multiple complications related to therapy. Euglycaemia was eventually achieved with PRRT.

## Patient consent

Written informed consent has been obtained from the patient for publication of the submitted article. The patient has been provided with a copy of the submitted work prior to consenting.

## Author contribution statement

S Abell and N Sachithanandan drafted the manuscript. All other authors have provided significant intellectual contribution to writing and revision of the manuscript. All authors were equally involved as physicians in the long-term care of the patient.
